# Sexual Arousal and Implicit and Explicit Determinants of Condom Use Intentions

**DOI:** 10.1007/s10508-018-1257-9

**Published:** 2018-07-12

**Authors:** Kenny Wolfs, Arjan E. R. Bos, Fraukje E. F. Mevissen, Gjalt-Jorn Y. Peters, Jacques J. D. M. van Lankveld

**Affiliations:** 1 Department of Psychology and Educational Sciences, Open University, Heerlen, The Netherlands; 2 Department of Work and Social Psychology, Maastricht University, Maastricht, The Netherlands; 3 Treatment Center for People with a Mild Intellectual Disability, AltraCura, 6161 DJ Geleen, The Netherlands

**Keywords:** Sexual risk, Sexual arousal, Attitudes, Implicit Association Test, Dual-process model

## Abstract

Being sexually aroused may be an important risk factor contributing to sexual decision making. Dual-process cognitive models, such as the reflective–impulsive model of Strack and Deutsch (2004), could be used to explain the effect of sexual arousal on intentions to use a condom. In this study, we investigated whether explicit and implicit attitudes toward condom use can predict intentions to use a condom when participants are sexually aroused and not aroused. In a within-subjects experimental design, male participants (*N* = 27) watched both a neutral and an erotic movie clip in counterbalanced order. After each clip, participants completed a questionnaire assessing their intentions to use a condom and explicit condom attitudes, followed by a wanting Implicit Association Test (IAT; Greenwald et al., 2003) and a liking IAT to assess their implicit attitudes to unsafe sex. In concordance with the reflective–impulsive model, we found that when participants were not sexually aroused, their intentions to use a condom were solely predicted by their explicit attitudes. However, when they were sexually aroused, intentions to use a condom were predicted by both explicit and implicit attitudes toward condom use.

## Introduction

According to the latest CDC report, nearly 20 million new sexually transmitted infections (STI) were reported in the U.S. in 2012 (Center for Disease Control and Prevention, 2013); the European CDPC reported 29306 new HIV infections in 2012 in Europe (European Centre for Disease Prevention and Control, 2014). Even though injecting drug users, sex workers, and men who have sex with men are mostly perceived as the groups at risk of attaining STIs, high rates of STI are also found among heterosexual young adults (UNAIDS, 2014). For successful development of interventions to promote condom use, we first need to establish the determinants of unsafe sexual behavior (Eldredge, Markham, Ruiter, Kok, & Parcel, 2016).

Though determinants of unsafe sexual risk behavior have been mostly studied from a cognitive perspective (e.g., Sheeran, Abraham, & Orbell, 1999), contextual factors such as sexual arousal may be an important predictor as well (Ariely & Loewenstein, 2006; George et al., 2009; MacDonald, Fong, Zanna, & Martineau, 2000; Norris et al., 2009; Skakoon-Sparling, Cramer, & Shuper, 2016). From these studies that experimentally investigated the effect of sexual arousal on intentions to engage in unprotected sex, the conclusion was drawn that when people are sexually aroused, their intentions to engage in unprotected sex become stronger. Sexual arousal thus seems to be an important determinant of sexual risk behavior and some have claimed that the effect of alcohol on sexual risk taking is completely mediated by sexual arousal (George et al., 2009; Norris et al., 2009). Sexual arousal is also known to increase sexual risk taking, as exhibited in studies using the Balloon Analog Risk Taking test (Prause & Lawyer, 2014), an approach-avoidance task (Simons, Maisto, Wray, & Emery, 2016), a Go/No-Go task (Macapagal, Janssen, Fridberg, Finn, & Heiman, 2011), and as reported in qualitative studies (Strong, Bancroft, Carnes, Davis, & Kennedy, 2005).

Given the importance of contextual factors such as sexual arousal in sexual risk taking, a theoretical framework that includes adaptability to contextual influences is necessary to accurately predict people’s behavior in different states. Dual-process models (Evans, 2003) include contextual determinants and thus seem to be apt to explain the effect of sexual arousal on sexual risk behavior. Dual-process models that focus on explicit or controlled as well as on implicit or automatic processes in decision making have attracted attention in health psychology research (Sheeran, Gollwitzer, & Bargh, 2013). According to these models, decision making is in part influenced by heuristic or fast-and-frugal reasoning strategies, which are based on implicit associations (Gilovich, Griffin, & Kahneman, 2002). Sniehotta, Presseau, and Araújo-Soares (2014) and Fishbein and Ajzen (2010) argued that implicit associations regarding condom use could be an important determinant of unsafe sex that should be taken into consideration. In the most recent version of the incentive-motivation model, Toates (2015) also included a fast-and-frugal system.

Thus far, however, research on applications of dual-process models (see Evans, 2003) to explain sexual risk taking has been scarce. Czopp, Monteith, Zimmerman, and Lynam (2004) studied the correlation between the IAT on intentions to use a condom in different conditions. In their study, participants were either exposed to a high-cue (meet a date at happy hour) or a low-cue scenario (dinner and a movie). A low-cue scenario was defined as a story in which there were no cues that would remind the participants of sexual risk, such as by describing that one has sex with one’s steady partner. A high-cue scenario, however, contained many cues pointing out a possibility to attain HIV, for example, by depicting a situation in which one has casual sex with someone whose serostatus is unknown. In the high-cue condition, explicit processes took over and were the only predictors of intentions to use a condom. In the low-cue condition, when behavior was more guided by automatic processes, implicit associations also predicted intentions to use a condom. Czopp et al. showed that implicit associations can predict condom use, but only under specific conditions. This study did not, however, induce different states in participants, such as, for example, a state of sexual arousal. Nevertheless, the investigators demonstrated the importance of implicit processes under specific conditions. Marsh, Johnson, and Scott-Sheldon (2001) corroborated the finding that implicit attitudes toward condom use predict condom use, but only for condom use with casual partners. In a steady relationship, participants’ condom use was best predicted by explicit attitudes.

Den Daas, Häfner, and de Wit (2014) investigated the effect of long-term health goals on sexual risk behavior in an impulsive versus a reflective state. They found that the effect of long-term health goals did not differ between both states. The studies by Den Daas et al. and Czopp et al. (2004) were among the first studies to test whether a dual-process model could be used to explain sexual risk behavior. These studies, however, did not investigate the effect of sexual arousal. In the present study, we therefore aimed to investigate whether implicit attitudes can account for (part of) the variance regarding the effect of sexual arousal on intentions to use a condom.

**Fig. 1:**
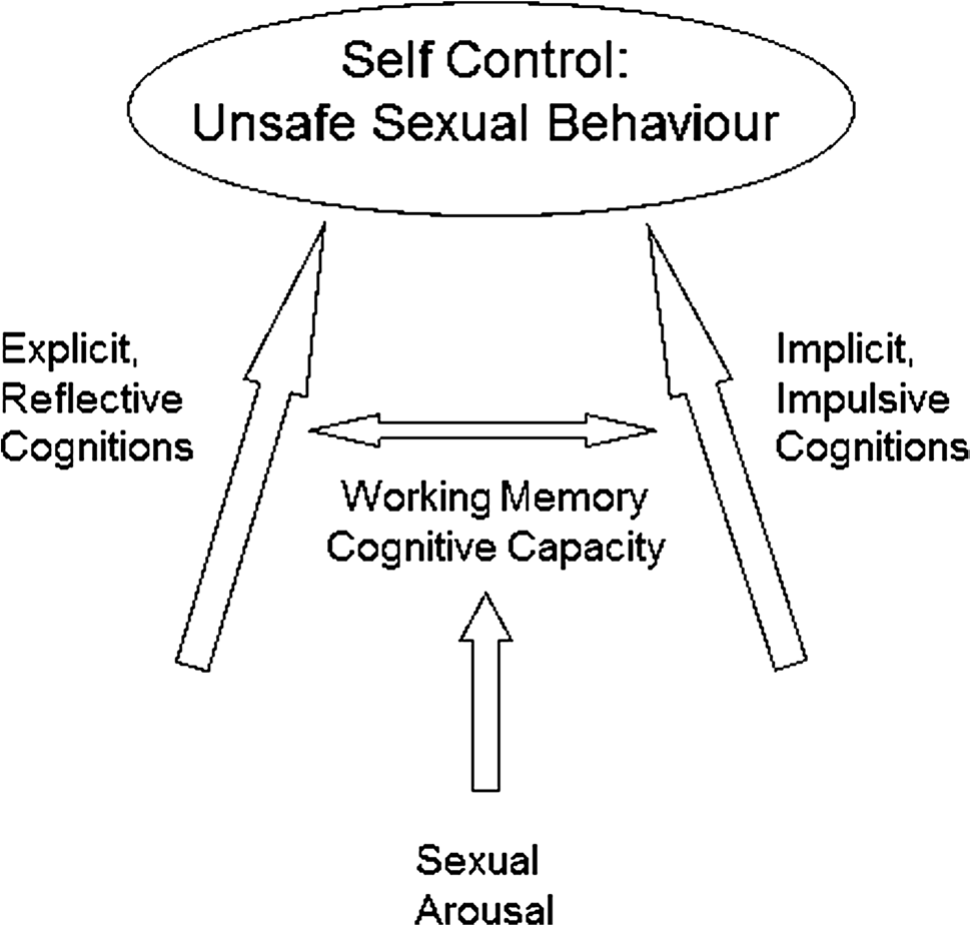
Reflective–impulsive model (Strack & Deutsch, 2004) adapted for sexual risk behavior due to sexual arousal

Our study was based on the reflective–impulsive model (RIM) of Strack and Deutsch (2004) (see Fig. 1). This model was chosen because it offers testable hypotheses, which are operationalized in readily measurable concepts, including working memory capacity. The model postulates that human decision making involves two parallel brain systems: a reflective system, which contains explicit cognitions, and an impulsive system, which contains implicit associations. Explicit attitudes and self-efficacy toward condom use, risk perception of attaining an STI, appraisals of negative consequences about not using a condom, and knowledge about STIs are examples of explicit cognitions that are part of the reflective system. The impulsive system is perceived as a simple and associative network. According to the RIM, the links between elements in the implicit system have different strengths and can only be gradually changed through learning. A positive implicit attitude about condoms, for example, implies that, when the concept of “condom” is activated, everything in this associative network related to the concept of “positive” is also activated. If the relationship between the concept of “condom” and “positive” is strong, the implicit attitude toward condoms is positive. The RIM postulates that the impulsive system may be accompanied by an experiential state of awareness, in which people might experience certain feelings or urges without knowing its origin. Therefore, the impulsive system may, but does not have to, operate outside of conscious awareness. The concept of implicit associations in the impulsive system shows overlap with the concept of sexual self-schema (Andersen, Cyranowski, & Espindle, 1999). Both sexual self-schemas and implicit associations are seen as associative networks of concepts. However, people are aware of their sexual self-schemas, while the impulsive system lies mainly outside of conscious awareness.

In this study, we decided to differentiate between implicit liking of condoms, as well as implicit wanting of condoms. The distinction between liking and wanting has also been questioned. Havermans (2011), for example, claimed that, based on research on eating disorders, there was no conceptual difference between implicit liking and wanting. However, liking and wanting were found to have distinct effects on sexual behavior (Dewitte, 2015). The study by Krishnamurti and Loewenstein (2012) gives further evidence that liking and wanting are two distinct features when it comes to sexuality. Based on the research in the field of sexology, we decided to include two different implicit association tests (IATs) that measured the concept of liking and wanting, respectively, with regard to condom use.

According to the RIM (Strack & Deutsch, 2004), the relative influence of the reflexive and the reflective system on human behavior is moderated by the available working memory capacity. When sufficient working memory capacity is available, the reflective system is able to override the automatic impulses of the impulsive system, thus constraining the impulsive system’s influence on behavior. However, when working memory capacity is reduced, behavior is less guided by the reflective system, and the impulsive system has a stronger influence on behavior. Individuals with low-trait working memory capacity have been found to show lower response inhibition in a Go/No-Go task (Finn, Justus, Mazas, & Steinmetz, 1999). Sexual stimuli were found to occupy working memory (Carvalho, Leite, Galdo-Álvarez, & Gonçalves, 2011), possibly because they are salient and capture attention (van Lankveld & Smulders, 2008).

A vital assumption when applying the RIM to sexual risk behavior is that people have more negative associations with condom use on an implicit level than on an explicit level. Czopp et al. (2004) found that implicit associations were slightly positive in a sample of college students. This does not contradict the RIM; implicit associations only have to be less positive than explicit attitudes for a shift in behavior to occur. To our knowledge, the assumption that implicit attitudes toward condoms are significantly more negative than explicit attitudes has not yet been investigated.

It is practically and ethically challenging to observe actual sexual risk behavior in a laboratory setting. Sexual risk behavior in an experimental setting has thus often been measured as people’s intentions to engage in unsafe sex (e.g., George et al., 2009; MacDonald et al., 2000). Intentions have a strong correlation with actual condom use according to a meta-analysis by Albarracin, Johnson, Fishbein, and Muellerleile (2001). Such intentions do not equal the actual behavior, but they are as close to it as one can get in a laboratory setting. We have therefore chosen to investigate the effect of sexual arousal using the explicit intention to use a condom as the outcome measure.

We tested the following hypotheses to explain the effect of sexual arousal on intentions to use a condom:

### **Hypothesis 1**

There will be a main effect of explicit attitudes on intentions to use a condom, which represents the effect attitudes have on intentions in a neutral state. Positive explicit attitudes toward condom use will be associated with stronger intentions to use a condom.

### **Hypothesis 2a**

There will be a moderating effect of sexual arousal on the effect of implicit attitudes regarding condom use on intentions to use a condom. Implicit attitudes will only show an effect on intentions to use a condom when participants are sexually aroused.

### **Hypothesis 2b**

An interaction effect of sexual arousal and explicit attitudes toward condom use is expected on the intention to use a condom. Explicit attitudes will have less impact on intention to use a condom when participants are sexually aroused.

### **Hypothesis 3**

On the implicit level, participants will show more negative attitudes toward safe sex than on the explicit level.

## Method

### Participants

All participants were psychology or medicine students at Maastricht University, participating for either student credit or a monetary fee of 25 euros. Inclusion criteria were: heterosexual men between 18 and 35 years old. We recruited a total of 32 male participants for this experiment. Five participants were excluded from further analysis: One participant turned out to not have a sufficient knowledge of the Dutch language to fully understand the questionnaires and the implicit association test. Two participants were rejected for not showing or reporting any sexual arousal during the erotic condition, and another was excluded because he showed far higher genital arousal in the neutral condition than in the erotic condition. A final participant was removed because he admitted not really to have understood the IAT, and not to have fully concentrated on the test either, as he was very tired. The remaining 27 male participants whose data were used in the analyses had a mean age of 21.7 (SD = 2.75), ranging from 18 to 30 years old. Most participants were Caucasian (*N* = 23; 85%). One participant was Asian; one was Middle Eastern; one described himself as Moroccan-Dutch, and one as Afro-Asian. Most men were in an ongoing relationship (*N* = 18; 67%). Information on the main outcome variables in this study is given in Table 1.

**Table 1:** Means and SD in the erotic and the neutral condition of sexual responses, explicit condom attitudes, and implicit condom attitudes

	Neutral		Erotic	
	*M*	SD	*M*	SD
Penile circumference (mm)	130.84	63.78	138.49	71.57
Subjective arousal (1–100)	5.13	8.63	46.44	21.70
Attitude: Wise (1–100)	82.66	12.24	80.31	12.13
Attitude: Pleasure (1–100)	34.19	24.72	33.34	25.49
Attitude: Easy (1–100)	54.72	22.39	50.34	34.41
Implicit wanting (D600)	− .07	.38	− .07	.38
Implicit liking (D600)	− .19	.36	− .28	.36

Ethical approval for the study was obtained from the Ethical Review Board of Zuyderland Medical Center in Heerlen, as well as the Maastricht University Ethics Research Committee Psychology and Neuroscience (ERCPN) of the faculty of Psychology and Neuroscience of Maastricht University, the Netherlands.

### Design

A within-subjects design was used including two experimental conditions: an erotic condition, in which sexual arousal was induced by having participants watch an erotic video, and a neutral condition, in which participants watched a neutral movie. Participants entered the conditions in counterbalanced order.

### Procedure

**Fig. 2:**
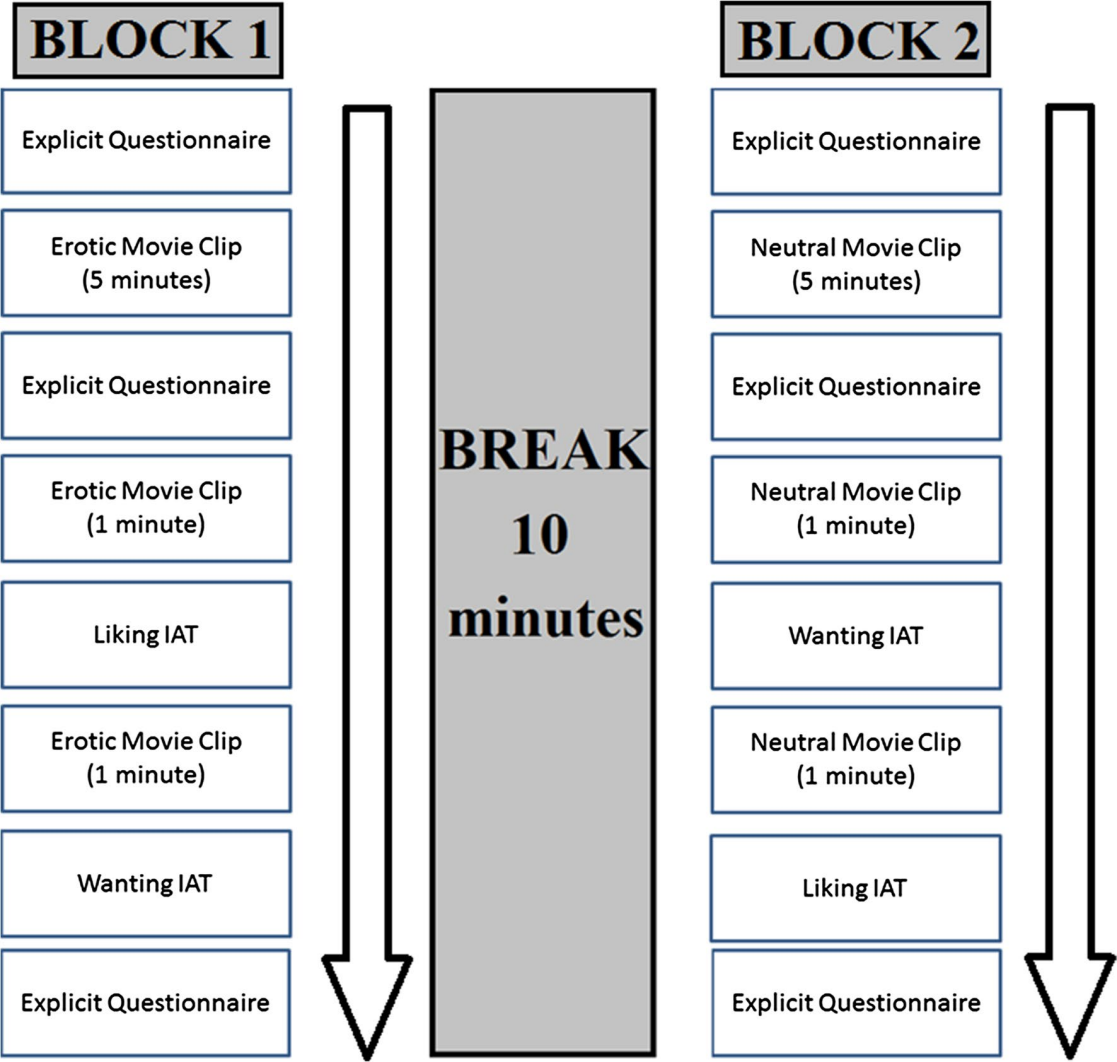
A visual depiction of our protocol, which consisted of two blocks and a break in between. All participants went through two blocks which were identical to each other except for the manipulation used. The order of the conditions, as well as the order in which the IATs were presented to participants, were counterbalanced

Participants entered the laboratory and first read and signed an informed consent form. To measure genital sexual arousal, a penile plethysmograph was used (Barlow, Becker, Leitenberg, & Agras, 1970). Instructions were given to participants on how to apply the penile plethysmograph themselves around the lower part of the penile shaft. After these instructions, the experimenter left the room and dimmed the lights in order to create a less clinical atmosphere. The light was kept at the same level for all participants. The experimenter was in an adjacent room to ensure privacy and could communicate with participants via an intercom system to give instructions. After participants had applied the plethysmograph, it was checked whether an adequate signal was produced. Next, participants completed a questionnaire, including the explicit measures (attitude and intention) and their current level of subjective sexual arousal. Participants then watched a video clip, which was either erotic or neutral in nature. This video was 5 min long in order to induce an adequate level of sexual arousal in the erotic condition, after which the questionnaire was repeated. Next, participants watched a 1-min (erotic or neutral) movie clip to ensure that sexual arousal remained high in the erotic condition. After this movie clip, participants performed both IATs. The question arises whether administering the explicit questionnaire might have influenced scores on the IAT. Research by Hofmann, Gawronski, Gschwendner, Le, and Schmitt (2005) suggests that it does not matter whether the explicit measure is administered before or after the IAT. Finally, participants once more completed the questionnaire to assess whether sexual arousal was maintained after performing two IATs. Participants now had a 10-min break in which they were asked to play some casual games on a computer tablet to try and wash out carry-over effects and to allow genital and subjective sexual arousal to return to baseline for those in the erotic condition. The penile plethysmograph was left in place during this break. After the break, participants entered the other condition. A graphical representation of the protocol is shown in Fig. 2.

### Material

#### Movie Clips

Four movie clips were used: two for the erotic and two for the neutral condition. Each condition had a 5-min and two 1-min clips. The erotic movies showed a heterosexual couple engaging in oral sex, and subsequently in (unprotected) vaginal penetration and were obtained from an online pornography site. The neutral movie clips were extracted from the BBC “Earth”-series. The 5-min neutral clip showed a documentary fragment about Antarctica, while the 1-min clips were taken from a documentary about “time-lapse filming techniques.” All movie clips were selected after pilot testing their ability to impact sexual arousal and have been previously used in our laboratory (e.g., Grauvogl et al., 2015).^1^

#### Manipulation Check: Genital Arousal

Genital sexual arousal was measured using an electromechanical strain gauge for penile circumference measurement (Barlow et al., 1970). This gauge was connected via a Wheatstone bridge to the Nexus-10 recording device (Mind Media BV, Roermond-Herten, Netherlands). The Nexus-10 is a mobile psychophysiological data acquisition system that can also be used to measure penile plethysmography. Data were recorded in mV, with a sampling rate of 32 per second. An average mV for each time point was calculated. After each participant was tested, the gauge was calibrated using a metal calibration cone with five subsequent steps of 5 mm. This allowed to convert the output to mm for further analysis. The gauge was disinfected in Cidex-OPA, a glutaraldehyde solution, for at least 10 min, rinsed with water and air-dried, before the arrival of each participant.

#### Manipulation Check: Subjective Arousal

Participants were asked to report their sexual arousal on a visual analog scale (VAS). This scale consists of a line of 100 mm, and ranges from 0 to 100 and represents a continuum between the statements “Not aroused at all” (= 0) and “Very aroused” (= 100). Participants placed a mark on the line where they felt their sexual arousal was situated on this continuum.

#### Outcome Measures: Explicit Cognitions

The explicit attitudes and intentions toward safe sex were measured after participants were first asked to read and imagine a situation in which they would meet a woman in a bar. The encounter leads up to the participant’s home, and there is an opportunity to have sex. While imagining this situation, participants answered attitude and intention questions using a 100-mm VAS ranging from 0 to 100. Condom use attitude was measured with three items (Cronbach’s *α* = .61): “How wise would it be to use a condom in this situation?” (*Very unwise*–*Very wise*), “How pleasant would it be to use a condom in this situation?” (*Very unpleasant*–*Very pleasant*), “How hard would it be to use a condom in this situation?” (*Very hard*–*Very easy*). We based these items on three main categories of attitudes regarding condom use, such as described in the Multidimensional Condom Attitude Scale (Helweg-Larsen & Collins, 1994). We also used studies regarding the barriers to condom use in Western college students to determine important dimensions of attitudes toward condom use for our study (Moore & Parker Halford, 1999; Wendt & Solomon, 1995). We chose to measure attitudes with this short scale because participants had to fill out this questionnaire several times throughout the experiment.

Condom use intention was measured with one item: “How much trust do you have that you would use a condom in this situation, even if the other party would not like to use one?” (*Very little trust*–*A lot of trust*). We wanted to avoid asking directly for intentions to use a condom in order to avoid socially accepted answers from participants. We had chosen this item based on a prestudy among 30 participants, in which it was found to show the highest correlation with a direct measure of intentions to use a condom, “I intend to use a condom in this scenario,” *r*(28) =* .*68*; p* < .001. We added the part about the other partner not wanting to use a condom to make the decision to use a condom more unilateral.

To avoid people remembering what they answered earlier on an item, we chose to measure intentions and explicit attitudes using a visual analog scale (VAS) over, for example, a Likert scale. The VAS has been found to be a reliable and valid way of measurement in the domain of pain assessment and management (Zalon, 1999), perceived quality of care (Arneill & Devlin, 2002), and psychoeducation (Dannon, Iancu, & Grunhaus, 2002).

#### Outcome Measures: Implicit Attitudes

Implicit condom attitudes were assessed using the implicit association test (Greenwald, Nosek, & Banaji, 2003). We constructed two versions of the IAT: a liking IAT and a wanting IAT (Dewitte, 2015; Dewitte & De Houwer, 2008; Tibboel et al., 2011; Wiers, Van Woerden, Smulders, & De Jong, 2002). Liking and wanting are seen as different concepts regarding other aspects of sexuality (Dewitte, 2015), and it may also be relevant to distinguish between these two concepts when it comes to safe sex. The liking IAT is a standard valence IAT, using “positive/negative” and “safe sex/unsafe sex” for the attribute and target categories. It was explained in the instructions that safe sex should be interpreted as sex with a condom, and unsafe sex should be interpreted as sex without a condom. They were also depicted as pictures of a penetration with either a condom or no condom present. The liking IAT represents a general implicit attitude toward safe sex. The wanting IAT is based on the personalized wanting IAT of Dewitte and De Houwer (2008). In this IAT, the categories were “I want/I do not want” and “safe sex/unsafe sex.” There were no right or wrong answers to the categories “I want/I do not want” since they were believed to be personal, and participants received no error feedback for these categories. The wanting IAT is said to capture a motivational process (Tibboel et al., 2011). In the study of Tibboel et al., implicit wanting correlated with the urge to smoke, demonstrating the predictive validity of the wanting IAT.

IATs consisted of five blocks. In the first block (18 trials), participants were asked to categorize words according to two attribute categories (e.g., positive/negative) that were displayed in the left and upper right corner of the computer screen. In the second block (congruent practice, 36 trials), participants categorized words into the same attribute categories, while a second target category of pictures was also introduced. Target labels were “safe sex” (penetration with a condom) and “unsafe sex” (penetration without a condom). The setup of the third block (congruent test, 48 trials) was similar to the second block. In the fourth block (incongruent practice, 36 trials), the positions of the attribute categories (e.g., safe sex/unsafe sex) on top of the screen were switched. In order to categorize words, participants now had to press the opposite keys of the ones they had to use in Blocks 1–3. The positions of “safe sex” and “unsafe sex” remained the same. The fifth (incongruent test) block (48 trials) had the same setup as the fourth block.

The words used for attributes are listed in Table 2, both in the original Dutch version and in the English translation. The pictures used for the attribute category “safe sex/unsafe sex” were taken from the Internet. All pictures depicted heterosexual vaginal penetration either with or without a visible condom. We only used pictures of penetration because it was necessary for participants to be able to identify a picture as either safe or unsafe sex as quickly as possible.^2^ We decided to use pictures for this category in order to keep sexual arousal high when participants were in the erotic condition, because we believed that an IAT task would act as a strong distractor and would diminish sexual arousal substantially. Performing non-sexual cognitive tasks is known to reduce sexual arousal (Geer & Fuhr, 1976; van Lankveld & van den Hout, 2004). The pictures were tested in an earlier pilot study for arousal, and three pictures from each category were chosen that received the highest arousal ratings. There might also be a risk of participants becoming aroused because of the IAT. However, we deliberately chose to use these “erotic” images for the IAT, because we believed the risk of losing sexual arousal during the IAT was much larger than the risk of becoming aroused by an IAT. The IAT was presented using OpenSesame (Mathôt, Schreij, & Theeuwes, 2012).

**Table 2:** Words used for the attributes in the implicit association test

	Liking IAT		Wanting IAT	
Dutch	Positief	Negatief	Wil ik graag	Wil ik niet graag
	Gezond	Haat	Verlangen	Vermijden
	Cadeau	Oorlog	Wensen	Ontwijken
	Vrede	Ziekte	Begeren	Afweren

English	Positive	Negative	I want	I do not want
	Healthy	Hate	Crave	Avoid
	Gift	War	Wish	Dodge
	Peace	Disease	Desire	Block

### Statistical Analysis

First, a check of the sexual arousal manipulation was performed, using paired-samples *t* tests of the difference in subjective as well as physiological arousal at several time points in the protocol.

IAT scores were calculated using the D600 algorithm (Greenwald et al., 2003) in which the mean reaction time during congruent trials (positive + safe sex) was subtracted from the mean during incongruent trials (negative + safe sex). All reaction times, responses both to word and to picture stimuli were used to calculate the mean reaction time in one set of trials. The minimum response time was set at 400 ms, and the maximum response time was set at 2500 ms. Any responses below this interval were omitted, while any responses above this interval were recoded to 2500 ms in accordance with Greenwald et al. (2003). Incorrect answers incurred a penalty of 600 ms, which was added to the observed reaction time. We then used these corrected reaction times to calculate the D600 score for the total IAT score (Blocks 2–5), for the practice trials (Blocks 2 and 4), and for the test trials (Blocks 3 and 5). The D600-scores were coded in such a way that a higher score on the IAT represented a more positive implicit attitude toward condom use for easier interpretation.

The first two hypotheses were tested using mixed regression analysis using an unstructured covariance matrix, random slope, and random intercept. Sexual arousal, the explicit attitude toward condom use, implicit condom attitude (IAT D600-score), and the interaction terms of these predictors with sexual arousal were entered as predictors in this regression model, using intention to use a condom as dependent variable. Two separate regression analyses were run using implicit liking and implicit wanting as predictor of intentions to use a condom, respectively.

To test our third hypothesis, the scores on the IATs and the VAS were rendered statistically comparable. We first subtracted 50 from the scores on the VAS for the explicit attitudes. This way, a score of 0 on both the VAS and the IAT implied a neutral stance toward safe sex. We then divided the scores of both the IAT as well as the VAS by their respective SD, rendering both outcomes independent of the scale they were measured on. Finally, the hypothesis was tested by running a paired-samples *t* test in both the erotic and the neutral condition between the liking IAT and the general attitude toward condom use. The same test was performed using the wanting IAT and the general attitude toward condom use.

## Results

### Sexual Arousal Manipulation Check

The means and SDs of genital and subjective arousal in both conditions are given in Table 2. The manipulation intended to induce sexual arousal was successful, both regarding subjective and physiological arousal. Penile circumference was significantly different during the erotic movie clip, compared to the neutral clip, *t*(26) = 2.34, *p* = .026. No significant difference in physiological arousal was observed during the first, *t*(26) = − 1.18, *p* = .25, or second IAT, *t*(26) <1. No significant baseline difference in subjective sexual arousal was found between the two conditions as measured in the first questionnaire, *t*(26) = 1.25, *p* = .22. Participants reported higher subjective sexual arousal in the erotic condition as compared to the neutral condition right after watching the 5-min movie, *t*(26) = 11.63, *p* < .001, as well as at the end of the condition after performance of both IAT tasks, *t*(26) = 3.73, *p* = .001. Means and SD of genital and subjective arousal scores are given in Table 2.

### Prediction of Condom Use Intention by Explicit and Implicit Condom Attitudes and Sexual Arousal

Table 3 shows the results of the mixed regression model using sexual arousal, explicit condom attitude, and implicit wanting as predictors of condom use intentions. Explicit attitudes significantly predicted intentions to use a condom (*B *= 1.38 *p* < .001). No other significant predictors were found. This finding corroborates our first hypothesis, whereas no empirical support can be found for our second hypothesis.

**Table 3:** Mixed regression model with intention to use a condom as a dependent variable and sexual arousal, implicit wanting, and explicit attitudes as predictors

Variable	*B*	SE B	*β*	*p*
Intercept	− 2.46	4.67		.013
Sexual arousal	− 1.48	2.35	− .05	.537
Explicit attitude	1.38	.21	.77	< .001
Implicit wanting	− 1.48	6.44	− .02	.820
Sexual arousal × explicit attitude	.02	.16	.01	.892
Sexual arousal × implicit wanting	9.20	6.56	.09	.176

Explicit attitudes and implicit wanting are centered around 0

Table 4 shows the results of the mixed regression model using sexual arousal, explicit condom attitude, and implicit liking instead of implicit wanting as predictors of condom use intentions. As predicted by hypothesis 1, explicit attitudes again significantly predicted intentions to use a condom (*B *= 1.39*; p* < .001). In addition, the interaction of sexual arousal and implicit liking predicted condom use intention (*B *= 17.11*; p* = .022). This implies that when participants were not sexually aroused, only explicit attitudes toward condoms significantly predicted their intentions to use a condom, but when participants were sexually aroused, implicit liking additionally predicted condom use intentions. No independent effects of sexual arousal or implicit liking were found.

**Table 4:** Mixed regression model with intention to use a condom as a dependent variable and sexual arousal, implicit liking, and explicit attitudes as predictors

Variable	*B*	SE B	*β*	*p*
Intercept	− 13.27	4.86		.011
Sexual arousal	1.80	2.47	− .04	.473
Explicit attitude	1.39	.02	.78	< .001
Implicit liking	− 6.13	9.21	− .07	.511
Sexual arousal × explicit attitude	.07	.15	.04	.633
Sexual arousal × implicit liking	17.11	6.93	.21	.022

Explicit attitudes and implicit liking are centered around 0

**Fig. 3:**
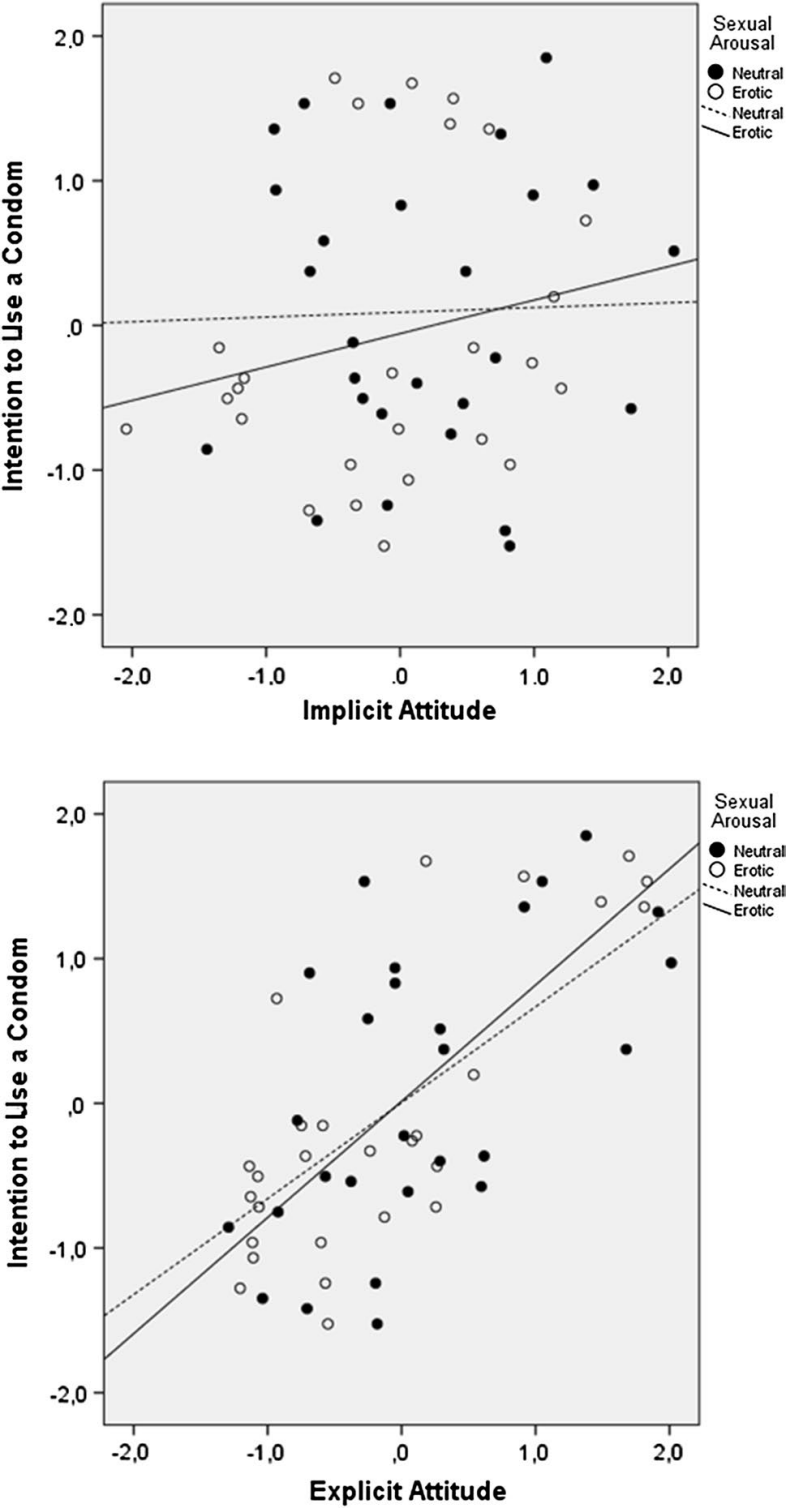
Implicit liking is related to intentions to use a condom, but only when participants are aroused. There is a strong effect of explicit attitudes on intentions to use a condom, both in the erotic as well as the neutral condition

A graphical representation of the effect of explicit attitudes and implicit liking on intentions to use a condom is shown in Fig. 3. To obtain the unique contribution of explicit attitudes (without the overlap of implicit attitudes), we first conducted a regression analysis with explicit attitudes (centered *z*-scores) as dependent variable and implicit attitudes (centered *z*-scores) as independent variable. The residuals were saved and represented the unique effect of explicit attitudes. The same was done to obtain the unique contribution of implicit attitudes, but now a regression analysis with implicit attitudes as the dependent variable and explicit attitudes as the independent variable was used. The values of “Intentions to use a condom” were also *z*-scores centered around zero.

Testing our third hypothesis, we found a difference between the implicit and explicit attitudes in both the neutral, *t*(26) = 3.54, *p* = .001, and the erotic condition, *t*(26) = 3.66, *p* < .001. In the erotic condition, the mean *z*-score for the explicit attitudes was .29, while the mean *z*-score for the implicit attitudes was − .08. In the neutral condition, the mean *z*-score for the explicit attitudes was .48, while the mean *z*-score for the implicit attitudes was − .05. Because the IAT was scored such that a negative score implies negative attitudes toward condom use, the implicit attitudes toward condom use were significantly more negative than the explicit attitudes. For the wanting IAT, we found no significant difference between the implicit and the explicit attitudes toward safe sex, *t*(26) = − 2.11, *p* = .05, in the neutral condition or in the erotic condition, *t*(26) < − 1.

## Discussion

In this study, we investigated a dual-process model of sexual risk taking, based on the reflective–impulsive model (Strack & Deutsch, 2004). This model predicts that when people are high on working memory capacity, their explicit cognitions and attitudes mainly determine their behavior. When they are low on working memory capacity (e.g., because they are sexually aroused), their behavior is determined to a larger extent by their implicit cognitions. In our study, we found that when participants were not sexually aroused, their intentions to use a condom were solely predicted by their explicit attitudes toward condom use (Hypothesis 2a). Our results also indicated that implicit attitudes were only related to intentions to use a condom when sexually aroused (Hypothesis 2a). We also expected to find an interaction effect between sexual arousal and explicit attitudes toward condom use (Hypothesis 2b). This interaction effect was not found, which may have been due to power limitations. A replication study might be necessary using more participants to see whether this interaction effect can be detected in a larger sample. Finally, we also expected implicit attitudes toward condom to be more negative than the explicit attitudes. We found that the men were indeed less positive toward condoms on an implicit level than when they were asked to report how positive they were about condoms in a survey (Hypothesis 3).

This study has some implications for future studies and the theoretical construct of a dual-process model of sexual risk taking. First of all, we found that implicit associations did not have an influence on intentions to use a condom in a neutral, unaroused state. This is in concordance with other theories regarding (sexual) decision making, such as the Theory of Planned Behavior (Ajzen, 1985) or the Reasoned Action Approach (Fishbein & Ajzen, 2010). However, the finding that, when sexually aroused, individuals’ intentions were predicted by both their explicit attitudes as well as their implicit attitudes toward condom use supports a dual-process model.

We did not, however, find any interaction between explicit attitudes and sexual arousal. This implies that the effect of explicit attitudes was equally strong in both the neutral and the erotic condition. This would not necessarily mean that a dual-process model should be rejected. Our results indicate that implicit attitudes toward condom use gain relative influence on intentions to use a condom when participants are sexually aroused, but without diminishing the influence of explicit attitudes.

The model of Strack and Deutsch (2004) does, however, assume that cognitive resources need to be available for the explicit attitudes to have an influence on intentions to use a condom. It is unclear to what degree sexual arousal was able to fully deplete cognitive resources. That sexual arousal depletes cognitive resources is an assumption, based on the studies of Carvalho et al. (2011) and van Lankveld and Smulders (2008). It is hard to estimate to which degree the sexual arousal we invoked in the laboratory, which probably had a ceiling effect due to the clinical setting, was able to reduce working memory capacity. This highlights the importance of also including a measure of working memory capacity in future research.

Our third hypothesis tested the model’s assumption that implicit attitudes were more negative than the explicit attitudes. The explicit attitudes were measured using a visual analog scale, which made it possible to determine a truly neutral score on the explicit attitudes (50 on a scale ranging from 0 to 100) as well as on the implicit attitudes (a D600 score of 0 on an IAT implies no preference for either safe sex or unsafe sex). Once more, we found only a significant difference on the liking IAT, and not on the wanting IAT. Compared with the wanting IAT, the liking IAT more closely resembles an implicit evaluation (Tibboel et al., 2011) about condom use and thus may be a better implicit equivalent of an explicit attitude (Ajzen, 1985), supporting the early recommendation that a liking IAT may be of more value for investigating safe sex than a wanting IAT as suggested by Tibboel et al. (2011). It should be noted that explicit and implicit attitudes by nature cannot be completely independent. According to the reflective–impulsive model (Strack & Deutsch, 2004), implicit associations are formed through gradual learning processes. With regard to condom use, these implicit associations are most likely formed through sexual experiences with or without condoms, as well as education about condom use. These same experiences, however, also form one’s explicit attitudes regarding condom use. This means that there is very likely some overlap between the implicit and explicit attitudes, which confounds the analyses between the two types of attitudes.

Future research might also investigate other predictors of sexual risk taking that are also known to reduce working memory capacity, including alcohol intoxication, given its known direct effect on intentions to engage in unprotected sex (Davis et al., 2016; MacDonald et al., 2000; Maisto, Carey, Carey, Gordon, & Schum, 2004; Maisto et al., 2004; Schacht et al., 2010; Simons, Maisto, Wray, & Emery, 2015; Stoner, George, Peters, & Norris, 2007; Wray, Simons, & Maisto, 2015). Alcohol intoxication was also found to reduce working memory capacity (Casbon, Curtin, Lang, & Patrick, 2003; Cromer, Cromer, Maruff, & Snyder, 2010; Fillmore, Ostling, Martin, & Kelly, 2009; Finn et al., 1999).

Support for a dual-process model of sexual decision making was found when using a liking version of the IAT, but not when using a wanting version of the IAT. When participants were sexually aroused, their implicit wanting associations did not have an effect on intentions to use a condom, whereas the implicit liking associations did. This could be interpreted to mean that condom use behavior is determined by the valence attributed to condom use, but not by the implicit motivation to use one (Tibboel et al., 2011). Similarly, implicit wanting associations did not differ between smokers who recently smoked or had been deprived of smoking for a few hours (Tibboel et al., 2011), whereas the liking IAT did show a difference between both groups, with the deprived group being more positive about cigarettes on the implicit level. Only few studies have focused on implicit wanting and liking with regards to sexual behavior. Dewitte (2015), for example, found that men’s sexual behavior in general (e.g., the frequency of coitus and/or masturbation) was best predicted by their implicit liking of sex, rather than their implicit wanting. For women, in contrast, implicit wanting predicted past sexual behavior rather than implicit liking. By analogy, men’s intentions to engage in unprotected sex when they are sexually aroused would be best predicted by implicit liking rather than by implicit wanting.

The present study had limitations that should be addressed in future research. A first assumption we have made in this research is that sexual arousal reduces working memory capacity. This assumption was made because results of previous research indicated that sexual stimuli automatically capture attention (van Lankveld & Smulders, 2008). People have less cognitive capacity left when they perform a second task, such as an auditory oddball task, while watching an erotic movie (Carvalho et al., 2011). Future research investigating sexual decision making, based on a dual-process model, should additionally measure working memory capacity to test this assumption. It might also be important to determine whether the effects of working memory capacity reduction differ on genital and subjective sexual arousal. Previous experimental research has shown that subjective sexual arousal has a stronger negative effect on intentions to use a condom (George et al., 2009; Norris et al., 2009).

Participants were not able to maintain high genital arousal while taking performing an IAT, but they did report a significantly higher subjective arousal at the end of the erotic condition compared to the neutral condition. The differential effect of reduced attention for erotic stimuli on, respectively, genital and subjective sexual arousal has been found in previous studies. The finding that genital arousal could not be maintained during a cognitive task, such as the IAT, is a finding that has been reported a long time ago by many authors (Geer & Fuhr, 1976; Salemink & van Lankveld, 2006; van Lankveld & van den Hout, 2004). It is thus not surprising that performing two IATs, taking approximately 5 min each, reduced genital arousal, even if they still subjectively felt sexually aroused.

Administering both a wanting and a liking IAT was, at the same time, a methodological weakness and strength of this study. The participants’ level of sexual arousal was not maintained during IAT performance, as shown in the manipulation check. To maintain a high level of sexual arousal, 1-min erotic video clips were presented before each IAT, but it proved not possible for participants to maintain their sexual arousal. Some of the choices made during the construction of the IATs might have caused validity problems. The target category labels (safe vs. unsafe sex) were kept as neutral as possible. However, the word unsafe in itself also might have a negative connotation. Another possible limitation relates to the recommendation by Nosek, Greenwald, and Banaji (2007) that categories should be defined as accurately as possible. We did so by mentioning in our instruction that “safe sex” should be interpreted as “sex with a condom” and “unsafe sex” as “sex without a condom.” Future research could also experiment using a single-target IAT. “Condoms” would seem a good target label that would avoid the dichotomy between safe and unsafe sex.

Even though the IAT has already been used in other studies regarding sexuality, such as those by Czopp et al. (2004) and Dewitte (2015), the IAT as a measure has received some criticism (Brendl, Markman, & Messner, 2001). More specifically, Blanton and Jaccard (2006) claim that although reaction time as an outcome measure in itself is non-arbitrary, deriving a magnitude of a preference for either safe or unsafe sex from these reaction times is. In other words, Blanton and Jaccard claim that no significant meaning regarding someone’s implicit preference can be attributed to reaction times on a computer task. Greenwald, Nosek, and Sriram (2006) responded to this criticism that the IAT is indeed a valuable measure because it has an absolute zero score, and that the scores are standardized. Nosek et al. (2007) reported that over 250 studies had already used the IAT as a measure at the time of their writing. Frequent use of a measure does not necessarily validate it, but in the current paradigm, the options for implicit measures which do not rely on introspection and are considered automatic are limited. Future studies could also experiment with other implicit measures such as the affective priming task (Krause, Back, Egloff, & Schmukle, 2012) or a Go/No-Go task (Eastwick, Eagly, Finkel, & Johnson, 2011; Macapagal et al., 2011).

Another limitation of this study was the very concise way of measuring explicit attitudes. Because of the repeated-measures design, we used a very brief assessment of participants’ explicit attitudes and intention toward condom use. Cronbach’s alpha for our attitude scale turned out to be sufficient, yet low. However, such a lower alpha can be expected in a scale with a limited number of items in a limited sample (Peterson, 1994). Intentions, moreover, were measured using a VAS response to a single item, to reduce the participants’ ability to remember their score on previous measurements. Future studies could use a multi-item measure of intention to use a condom.

In conclusion, implicit associations did not influence intentions to use a condom in a neutral state, but when participants were sexually aroused, implicit associations explained part of the variance of their intentions to use a condom. People were also more negative about condom use on an implicit level than on an explicit level. Most of the hypotheses we proposed based on an application of the reflective–impulsive model by Strack and Deutsch (2004) were corroborated. It is therefore important to further investigate the role of implicit factors in sexual risk taking and gain more insight into how they determine this behavior.

## Footnotes

1 The movie clips are available upon request from the corresponding author.

2 These pictures are available upon request from the corresponding author.

## References

[CR1] Ajzen I (1985). From intentions to actions: A theory of planned behavior.

[CR2] Albarracin D, Johnson BT, Fishbein M, Muellerleile PA (2001). Theories of reasoned action and planned behavior as models of condom use: A meta-analysis. Psychological Bulletin.

[CR3] Andersen BL, Cyranowski JM, Espindle D (1999). Men”s sexual self-schema. Journal of Personality and Social Psychology.

[CR4] Ariely D, Loewenstein G (2006). The heat of the moment: The effect of sexual arousal on sexual decision making. Journal of Behavioral Decision Making.

[CR5] Arneill AB, Devlin AS (2002). Perceived quality of care: The influence of the waiting room environment. Journal of Environmental Psychology.

[CR6] Barlow D, Becker R, Leitenberg H, Agras W (1970). A mechanical strain gauge for recording penile circumference change. Journal of Applied Behavior Analysis.

[CR8] Blanton H, Jaccard J (2006). Arbitrary metrics in psychology. American Psychologist.

[CR9] Brendl CM, Markman AB, Messner C (2001). How do indirect measures of evaluation work? Evaluating the inference of prejudice in the Implicit Association Test. Journal of Personality and Social Psychology.

[CR10] Carvalho S, Leite J, Galdo-Álvarez S, Gonçalves ÓF (2011). Psychophysiological correlates of sexually and non-sexually motivated attention to film clips in a workload task. PLoS ONE.

[CR11] Casbon TS, Curtin JJ, Lang AR, Patrick CJ (2003). Deleterious effects of alcohol intoxication: Diminished cognitive control and its behavioral consequences. Journal of Abnormal Psychology.

[CR12] Center for Disease Control and Prevention. (2013). *Incidence, prevalence and cost of sexually transmitted infections in the United States.* Retrieved January 14, 2016 from http://www.cdc.gov/std/stats/sti-estimates-fact-sheet-feb-2013.pdf.

[CR13] Cromer JR, Cromer JA, Maruff P, Snyder PJ (2010). Perception of alcohol intoxication shows acute tolerance while executive functions remain impaired. Experimental and Clinical Psychopharmacology.

[CR14] Czopp AM, Monteith MJ, Zimmerman RS, Lynam DR (2004). Implicit attitudes as potential protection from risky sex: Predicting condom use with the IAT. Basic and Applied Social Psychology.

[CR15] Dannon P, Iancu I, Grunhaus L (2002). Psychoeducation in panic disorder patients: Effect of a self-information booklet in a randomized, masked-rater study. Depression and Anxiety.

[CR16] Davis KC, Danube CL, Neilson EC, Stappenbeck CA, Norris J, George WH, Kajumulo KF (2016). Distal and proximal influences on men’s intentions to resist condoms: Alcohol, sexual aggression history, impulsivity, and social-cognitive factors. AIDS and Behavior.

[CR100] Den Daas C, Häfner M, de Wit J (2014). The impact of long-term health goals on sexual risk decisions in impulsive and reflective cognitive states. Archives of Sexual Behavior.

[CR17] Dewitte M (2015). Gender differences in liking and wanting sex: Examining the role of motivational context and implicit versus explicit processing. Archives of Sexual Behavior.

[CR18] Dewitte M, De Houwer J (2008). Adult attachment and attention to positive and negative emotional face expressions. Journal of Research in Personality.

[CR19] Eastwick PW, Eagly AH, Finkel EJ, Johnson SE (2011). Implicit and explicit preferences for physical attractiveness in a romantic partner: A double dissociation in predictive validity. Journal of Personality and Social Psychology.

[CR7] Eldredge, L. K. B., Markham, C. M., Ruiter, R. A., Kok, G., & Parcel, G. S. (2016). *Planning health promotion programs: An intervention mapping approach*. John Wiley & Sons.

[CR20] Evans JSB (2003). In two minds: Dual-process accounts of reasoning. Trends in Cognitive Sciences.

[CR22] Fillmore MT, Ostling EW, Martin CA, Kelly TH (2009). Acute effects of alcohol on inhibitory control and information processing in high and low sensation-seekers. Drug and Alcohol Dependence.

[CR23] Finn PR, Justus A, Mazas C, Steinmetz JE (1999). Working memory, executive processes and the effects of alcohol on Go/No-Go learning: Testing a model of behavioral regulation and impulsivity. Psychopharmacology.

[CR24] Fishbein M, Ajzen I (2010). Predicting and changing behavior.

[CR25] Geer JH, Fuhr R (1976). Cognitive factors in sexual arousal: The role of distraction. Journal of Consulting and Clinical Psychology.

[CR26] George WH, Davis KC, Norris J, Heiman JR, Stoner SA, Schacht RL, Hendershot CS, Kajumulo KF (2009). Indirect effects of acute alcohol intoxication on sexual risk-taking: The roles of subjective and physiological sexual arousal. Archives of Sexual Behavior.

[CR27] Gilovich T, Griffin D, Kahneman D (2002). Heuristics and biases: The psychology of intuitive judgment.

[CR28] Grauvogl A, de Jong P, Peters M, Evers S, van Overveld M, van Lankveld J (2015). Disgust and sexual arousal in young adult men and women. Archives of Sexual Behavior.

[CR29] Greenwald AG, Nosek BA, Banaji MR (2003). Understanding and using the Implicit Association Test: I. An improved scoring algorithm. Journal of Personality and Social Psychology.

[CR30] Greenwald AG, Nosek BA, Sriram N (2006). Consequential validity of the Implicit Association Test: Comment on Blanton and Jaccard (2006). American Psychologist.

[CR31] Havermans RC (2011). “You say it’s liking, I say it’s wanting…”: On the difficulty of disentangling food reward in man. Appetite.

[CR32] Helweg-Larsen M, Collins BE (1994). The UCLA Multidimensional Condom Attitudes Scale: Documenting the complex determinants of condom use in college students. Health Psychology.

[CR33] Hofmann W, Gawronski B, Gschwendner T, Le H, Schmitt M (2005). A meta-analysis on the correlation between the Implicit Association Test and explicit self-report measures. Personality and Social Psychology Bulletin.

[CR34] Krause S, Back MD, Egloff B, Schmukle SC (2012). A new reliable and valid tool for measuring implicit self-esteem: The Response-Window Affective Priming Task. European Journal of Psychological Assessment.

[CR35] Krishnamurti T, Loewenstein G (2012). The Partner-Specific Sexual Liking and Sexual Wanting Scale: Psychometric properties. Archives of Sexual Behavior.

[CR36] Macapagal KR, Janssen E, Fridberg DJ, Finn PR, Heiman JR (2011). The effects of impulsivity, sexual arousability, and abstract intellectual ability on men’s and women’s Go/No-Go task performance. Archives of Sexual Behavior.

[CR37] MacDonald TK, Fong GT, Zanna MP, Martineau AM (2000). Alcohol myopia and condom use: Can alcohol intoxication be associated with more prudent behavior?. Journal of Personality and Social Psychology.

[CR38] Maisto SA, Carey MP, Carey KB, Gordon CM, Schum JL (2004). Effects of alcohol and expectancies on HIV-related risk perception and behavioral skills in heterosexual women. Experimental and Clinical Psychopharmacology.

[CR39] Maisto SA, Carey MP, Carey KB, Gordon CM, Schum JL, Lynch KG (2004). The relationship between alcohol and individual differences variables on attitudes and behavioral skills relevant to sexual health among heterosexual young adult men. Archives of Sexual Behavior.

[CR40] Marsh KL, Johnson BT, Scott-Sheldon LA (2001). Heart versus reason in condom use: Implicit versus explicit attitudinal predictors of sexual behavior. Zeitschrift für Experimentelle Psychologie.

[CR41] Mathôt S, Schreij D, Theeuwes J (2012). OpenSesame: An open-source, graphical experiment builder for the social sciences. Behavior Research Methods.

[CR42] Moore S, Parker Halford A (1999). Barriers to safer sex beliefs and attitudes among male and female adult heterosexuals across four relationship groups. Journal of Health Psychology.

[CR43] Norris J, Stoner SA, Hessler DM, Zawacki T, Davis KC, George WH, Morrison DM, Parkhill MR, Abdallah DA (2009). Influences of sexual sensation seeking, alcohol consumption, and sexual arousal on women’s behavioral intentions related to having unprotected sex. Psychology of Addictive Behaviors.

[CR44] Nosek BA, Greenwald AG, Banaji MR, Bargh JA, Bargh JA (2007). The Implicit Association Test at age 7: A methodological and conceptual review. Social psychology and the unconscious: The automaticity of higher mental processes.

[CR45] Peterson RA (1994). A meta-analysis of Cronbach’s coefficient alpha. Journal of Consumer Research.

[CR46] Prause N, Lawyer S (2014). Specificity of reinforcement for risk behaviors of the Balloon Analog Risk Task using math models of performance. Journal of Risk Research.

[CR200] Salemink E, van Lankveld JJ (2006). The effects of increasing neutral distraction on sexual responding of women with and without sexual problems. Archives of Sexual Behavior.

[CR47] Schacht RL, George WH, Davis KC, Heiman JR, Norris J, Stoner SA, Kajumulo KF (2010). Sexual abuse history, alcohol intoxication, and women’s sexual risk behavior. Archives of Sexual Behavior.

[CR48] Sheeran P, Abraham C, Orbell S (1999). Psychosocial correlates of heterosexual condom use: A meta-analysis. Psychological Bulletin.

[CR49] Sheeran P, Gollwitzer PM, Bargh JA (2013). Nonconscious processes and health. Health Psychology.

[CR50] Simons JS, Maisto SA, Wray TB, Emery NN (2015). Acute effects of intoxication and arousal on approach/avoidance biases toward sexual risk stimuli in heterosexual men. Archives of Sexual Behavior.

[CR51] Skakoon-Sparling S, Cramer KM, Shuper PA (2016). The impact of sexual arousal on sexual risk-taking and decision-making in men and women. Archives of Sexual Behavior.

[CR52] Sniehotta FF, Presseau J, Araújo-Soares V (2014). Time to retire the theory of planned behaviour. Health Psychology Review.

[CR53] Stoner SA, George WH, Peters LM, Norris J (2007). Liquid courage: Alcohol fosters risky sexual decision-making in individuals with sexual fears. AIDS and Behavior.

[CR54] Strack F, Deutsch R (2004). Reflective and impulsive determinants of social behavior. Personality and Social Psychology Review.

[CR55] Strong DA, Bancroft J, Carnes LA, Davis LA, Kennedy J (2005). The impact of sexual arousal on sexual risk-taking: A qualitative study. Journal of Sex Research.

[CR57] Tibboel H, De Houwer J, Spruyt A, Field M, Kemps E, Crombez G (2011). Testing the validity of implicit measures of wanting and liking. Journal of Behavior Therapy and Experimental Psychiatry.

[CR58] Toates F, Gruber CW, Clark MG, Klempe SH, Valsiner J (2015). A grand synthesis: Aided by considering systems 1 and 2 and incentive motivation. Constraints of agency: Explorations of theory in everyday life.

[CR59] UNAIDS (2014). Gap Report.

[CR60] van Lankveld JJDM, Smulders FT (2008). The effect of visual sexual content on the event-related potential. Biological Psychology.

[CR61] van Lankveld JJDM, van den Hout MA (2004). Increasing neutral distraction inhibits genital but not subjective sexual qrousal of sexually functional and dysfunctional men. Archives of Sexual Behavior.

[CR63] Wendt SJ, Solomon LJ (1995). Barriers to condom use among heterosexual male and female college students. Journal of American College Health.

[CR64] Wiers RW, van Woerden N, Smulders FT, de Jong PJ (2002). Implicit and explicit alcohol-related cognitions in heavy and light drinkers. Journal of Abnormal Psychology.

[CR65] Wray TB, Simons JS, Maisto SA (2015). Effects of alcohol intoxication and autonomic arousal on delay discounting and risky sex in young adult heterosexual men. Addictive Behaviors.

[CR66] Zalon, M. L. (1999). Comparison of pain measures in surgical patients. *Journal of Nursing Measurement, 7*, 135–152. 10710858

